# Sustained heavy drinking over 25 years is associated with increased N-terminal-pro-B-type natriuretic peptides in early old age: Population-based cohort study

**DOI:** 10.1016/j.drugalcdep.2020.108048

**Published:** 2020-07-01

**Authors:** Annie Britton, Dara O’Neill, Diana Kuh, Steven Bell

**Affiliations:** a Department of Epidemiology and Public Health, University College London, London, WC1E 6BT, UK; b CLOSER, UCL Institute of Education, University College London, London, UK; c MRC Unit for Lifelong Health and Ageing at University College London, London, UK; d Department of Public Health and Primary Care, University of Cambridge, Strangeways Research Laboratory, Wort’s Causeway, Cambridge, CB1 8RN, UK; e Department of Clinical Neurosciences, University of Cambridge, Cambridge Biomedical Campus, Cambridge, CB2 0QQ, UK

**Keywords:** Alcohol, Hear failure, Cohort study

## Abstract

Heavy alcohol consumption is associated with an increased risk of heart failure. We sought to investigate whether levels of NT-proBNP differ by alcohol consumption profiles, both current drinking as well as cumulative exposure to drinking over several decades in a general population sample.

**Methods:**

Data on 2054 participants (49% male) were taken from the UK Medical Research Council National Survey for Health and Development, a longitudinal cohort study based on a nationally representative sample of births in 1946. Categories of long-term alcohol consumption were created based on consumption over 25 years of observations and compared with levels of NT-proBNP measured at mean age 63.

**Results:**

We found that those who drank heavily (both currently and long-term) had higher levels of NT-proBNP than moderate drinkers, after adjusting for major confounders (age, sex, socio-economic position and smoking). As NT-proBNP has attracted attention as a biomarker for heart failure, this suggests a critical pathway through which heavy drinking may increase risk of this cardiovascular disease. When we looked at heavy drinkers who varied their intake over the decades, it was only the recently heavy group that had higher levels of NT-proBNP. Further work is needed to demonstrate whether effects are reversible upon cessation of heavy drinking, but this finding highlights the need to have repeated data to unpack dynamics over time.

**Conclusion:**

Our findings suggest heavy drinkers could be screened for NT-proBNP levels in order to identify those at high risk earlier in the clinical stages of heart failure and targeted for risk reduction strategies.

## Highlights

• Prolonged heavy drinkers had higher levels of NT-proBNP, an important cardiac biomarker for heart failure, than moderate drinkers.
• This suggests a pathway through which prolonged heavy alcohol consumption may increase risk of this cardiovascular disease.
• Heavy drinkers could be screened for NT-proBNP levels to identify those at high risk earlier in the clinical stages of heart failure and targeted for risk reduction strategies.

## 1 Background

Heart failure (HF) affects 2% of the population in the developed world and is the single main cause of hospitalisations (Lund et al., 2018). This intensifies the need to study modifiable risk factors such as heavy alcohol consumption that has been shown to be associated with an increased risk of cardiovascular disease (Fernández-Solà, 2015), including elevated HF risk (Bell et al., 2017a, 2017b).

The effects of alcohol on the cardiovascular system are complex (Mathews et al., 2015). One aspect to note is that drinking is a dynamic behaviour that changes over the life course (Britton et al., 2015) and as such it is necessary to study the effects of longer term drinking exposure (Britton et al., 2016. The drinking habits adopted in early adulthood and middle age (a time typically free from disease) correlate with risk factors for cardiovascular disease (Britton et al., 2016) Thus, there is a need to be proactive in identifying those at risk early/before symptoms necessarily manifest (Bell, 2018).

Prospective studies in the general population have reported strong associations between circulating concentrations of N-terminal-pro-B-type natriuretic peptides (NT-proBNP), a marker of myocyte stress, and adverse cardiovascular outcomes including heart failure (Natriuretic Peptides Studies Collaboration, 2016). We therefore sought to investigate whether levels of NT-proBNP differ by alcohol consumption profiles, both current drinking as well as cumulative exposure to drinking over several decades in a general population sample. The purpose being that this might reveal a possible aetiological pathway between alcohol consumption and risk of heart failure, but also importantly highlight whether accounting for long-term alcohol intake is reflected in differences in levels of this blood biomarker and could therefore potentially be used to identify those at high risk earlier in the clinical stages of heart failure than current clinical practice. As alcohol-induced heart damage usually occurs with long-term alcohol misuse (Guzzo-Merello et al., 2014), we looked at consumption over a 25-year period.

### 1.1 Aims

To explore the relationship between alcohol consumption and NT-proBNP levels in a general population study over 25 years, accounting for longitudinal variability in consumption and other confounding factors.

## 2 Methods

### 2.1 Data

Data were available for 2054 participants (49% male) from the UK Medical Research Council National Survey for Health and Development (NSHD). The NSHD is a nationally representative sample of 5362 singleton births to married parents in England, Scotland and Wales, in 1 week in March 1946 (Kuh et al., 2001). The sample has been followed up repeatedly over seven decades. Written informed consent was provided by participants at each visit.

### 2.2 Measures

At ages 36, 43, 53 and 60–64, alcohol consumption was assessed with a 5-day food diary from which an estimate of total alcohol consumed per week was derived. Categories of long-term alcohol consumption were then created based on UK guidelines for ‘sensible drinking’ at the time of data collection (Department of Health Great Britain, 1995; O’Neill et al., 2018). These were: consistent non-drinkers, consistent moderate drinkers, consistent heavy drinkers, inconsistent moderate drinkers, inconsistent heavy drinkers and former drinkers. Moderate consumption was defined as ethanol intake to 112 g (≤14 UK units) for women and 168 g (≤21 UK units) for men per week (UK ‘sensible drinking’ in 1985). Heavy drinking was defined as consumption over these amounts. Drinkers with inconsistent levels of alcohol intake were defined initially according to their modal level of intake and then in a secondary analysis according to their most recent level of intake to account for shorter-term effects of particular consumption levels.

A cross sectional categorisation of drinking using intake at a single time point (age 60–64) was also employed with drinkers grouped as current heavy, moderate or non-drinkers using the same thresholds as described above.

NT-proBNP was measured at age 60–64 from venous blood samples (Kuh et al., 2019. Fasting overnight blood samples were taken by nurses, initially processed at clinical research facility laboratories, and stored at −80 °C. Frozen aliquots were transferred monthly to the MRC Human Nutrition Research laboratory in Cambridge. Analyses of NT-proBNP by automated electrochemiluminescence immunoassay were subsequently carried out at R&D Systems (Abingdon, UK)

Socio-economic position (categorised as low, intermediate or high, based on occupational status and retirement class data) and smoking status (categorised as non-smoker, current smoker, or ex-smoker) were self-declared within questionnaires at age 60–64.

### 2.3 Analysis

Quantile (median) regression was used to investigate whether drinker types differed in their associations with levels of NT-proBNP as the latter had a highly skewed distribution. Consistent moderate drinkers were used as the reference category in the longitudinal analyses, and current moderate drinking was used in the cross-sectional analyses. Adjustment was made in all models for sex, age, smoking, and socio-economic position, and interaction effects were also examined for sex and drinker type. Due to missing data, findings from complete and imputed datasets were compared. The latter comprised 100 datasets multiply imputed using chained equations. The statistical analyses were performed in Stata (v15.1; College Station, TX: StataCorp LLC). All statistical significance testing was two-tailed, using an inference threshold of p < 0.05.

## 3 Results

The mean age of participants was 63.3 years (SD = 1.1) at the time of the blood draw and the median value of NT-proBNP was 55.0 pg/mL (25th–75th percentiles = 31.0–95.0 pg/mL).

A higher proportion of consistent heavy drinkers were males (88% males) compared to consistent non-drinkers (27% men) (Table 1). Heavy drinkers were the least likely to be non-smokers (9%) whilst non-drinkers were most likely to be non-smokers (42%). By the mean age of 63 years, the proportion of ex-smokers was high, with heavy drinkers (both consistent and inconsistent) being the most likely to be ex-smokers (70% and 60%, respectively). Non-drinkers had the highest proportion of participants in the lowest socio-economic position (26%) and consistent moderate drinkers the least (7%).

**Table 1:** Drinker type definitions with observed counts and descriptive statistics at age 60–64.

Variable	Category	Consistent non-drinker	Consistent moderate drinker	Consistent heavy drinker	Inconsistent moderate drinker	Inconsistent heavy drinker	Former drinker	Unknown	All
		0 g at each wave of data collection	Male: 1−168 g per week, Females: 1−112 g per week at each wave	Male >168 g and female >112 g at each wave	Male: 1−168 g, Female: 1−112 g for most but not all waves	Male >168 g female>112 g for most but not all waves	0 g at last wave but intake >0 g at any earlier wave		
Sex	Male	39 (27.5%)	285 (48.1%)	58 (87.9%)	268 (47.7%)	157 (65.7%)	120 (38.1%)	70 (50.7%)	997 (48.5%)
	Female	103 (72.5%)	307 (51.9%)	8 (12.1%)	294 (52.3%)	82 (34.3%)	195 (61.9%)	68 (49.3%)	1057 (51.5%)
Age, mean (SD)		63.5 (1.08)	63.3 (1.12)	63.1 (1.16)	63.2 (1.17)	63.2 (1.21)	63.5 (1.03)	63.4 (1.07)	63.3 (1.13)
Smoking status	Non-smoker	60 (42.3%)	213 (34.0%)	6 (9.1%)	152 (27.1%)	49 (20.5%)	89 (28.3%)	34 (24.64)	603 (29.4%)
	Current smoker	16 (11.3%)	42 (7.1%)	8 (12.12)	46 (8.2%)	33 (13.8%)	43 (13.7%)	23 (16.7%)	211 (10.3%)
	Ex-smoker	54 (38.0%)	292 (49.3%)	46 (69.7%)	324 (57.7%)	143 (59.8%)	152 (48.3%)	54 (39.13)	1065 (51.9%)
	Missing	12 (8.5%)	45 (7.6%)	6 (9.1%)	40 (7.1%)	14 (5.9%)	31 (9.8%)	27 (19.6%)	175 (8.5%)
Socio-economic position	High	44 (31.0%)	327 (55.2%)	42 (63.6%)	285 (50.7%)	134 (56.1%)	122 (38.7%)	51 (37.0%)	1005 (48.9%)
	Intermediate	57 (40.1%)	221 (37.3%)	17 (25.8%)	204 (36.3%)	82 (34.3%)	145 (46.0%)	60 (43.5%)	786 (38.3%)
	Low	37 (26.1%)	44 (7.4%)	7 (10.6%)	73 (13.0%)	23 (9.6%)	48 (15.2%)	19 (13.8%)	251 (12.2%)
	Missing	4 (2.8%)	0 (0.0%)	0 (0.0%)	0 (0.0%)	0 (0.0%)	0 (0.0%)	8 (5.8%)	12 (0.6%)

**Fig. 1:**
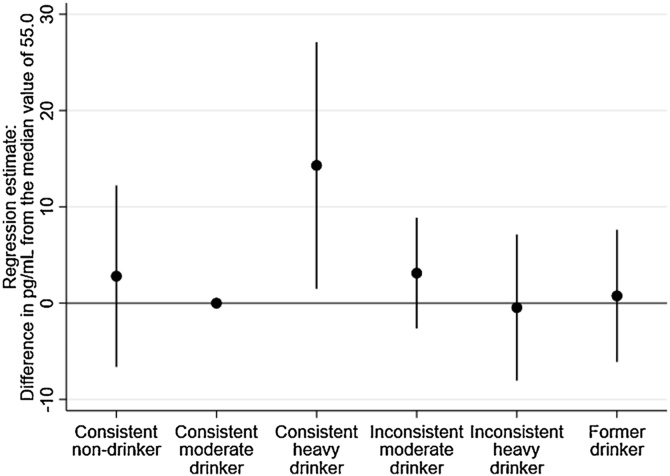
Association between long-term alcohol consumption drinker types and NT-proBNP in 2054 adults aged 60–64 using quintile regression analysis. Adjusted for age, sex, socioeconomic position and smoking (imputed data shown, similar association observed when restricted to complete case analysis).

Men and women were combined for the regression analyses as there was no evidence of an interaction effect (see supplementary materials). In analyses based on imputed data (see Fig. 1), consistent heavy drinkers were found to have significantly higher levels of NT-proBNP compared to consistent moderate drinkers after adjustment for sex, age, socioeconomic position and smoking status (β = 14.3; 95%CI = 1.5–27.1). All other drinking types showed no significant association with levels of NT-proBNP. In the complete case analysis (based on data from 1765 participants), a similar pattern of results was obtained, with the largest and only significant effect again observed for consistent heavy drinkers (see supplementary materials).

**Fig. 2:**
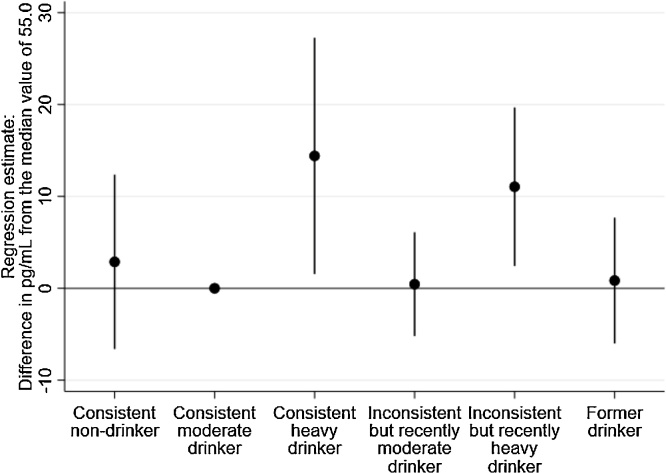
Association between current alcohol consumption drinker types (with inconsistent drinker types categorised by most recent intake levels) and NT-proBNP in 2054 adults aged 60–64 using quintile regression analysis. Adjusted for age, sex, socioeconomic position and smoking (imputed data shown, similar association observed when restricted to complete case analysis).

Modelling the alternative categorisation of drinker type that distinguished between those with recent moderate or heavy intake but with previously inconsistent levels showed that there were significant effects for both consistently heavy drinkers (β = 14.4, 95%CI = 1.6–27.3) and inconsistent but recently heavy drinkers (β = 11.1, 95%CI = 2.4–19.7) when compared to consistently moderate drinkers. The results are presented in Fig. 2.

**Fig. 3:**
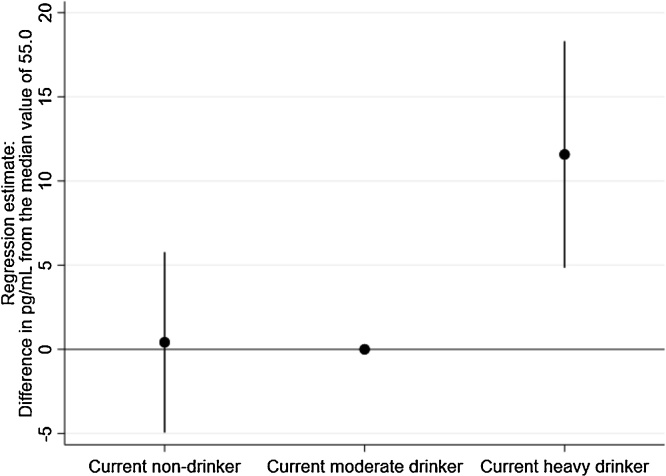
Cross sectional association between current alcohol consumption drinker types and NT-proBNP in 2054 adults aged 60–64 using quintile regression analysis. Adjusted for age, sex, socioeconomic position and smoking (imputed data shown, similar association observed when restricted to complete case analysis.

Cross-sectional analyses were run with drinker type defined according to a single intake assessment that occurred at the same time as the NT-proBNP measurement (Fig. 3). Participants reporting current heavy alcohol intake had significantly higher levels of NT-proBNP compared to participants currently reporting moderate levels of drinking (11.6, 95% CI = 4.8–18.3). This was found for both the imputed and complete case analyses. No difference was found between currently moderate drinkers and abstainers. Complete tabulation of the regression results for all models are presented in the supplementary materials.

## 4 Conclusion

We found that those who drank heavily over the 25-year observation period, had higher levels of NT-proBNP than moderate drinkers, after adjusting for major confounders (age, sex, socioeconomic position and smoking). As NT-proBNP has attracted attention as a biomarker for heart failure, this suggests a critical pathway through which prolonged heavy alcohol consumption may increase risk of this cardiovascular disease.

Our findings concur with those in a study of people with alcohol dependency where NT-proBNP was shown to be raised (Höfer et al., 2011). However, there are few population studies that have measured this association and none, as far as we are aware, that report long term alcohol consumption typologies and levels of these natriuretic peptides. Cross-sectional data from two population-based studies (one Russian and one UK) found that heavy and hazardous drinking were associated with elevated levels of B-type natriuretic peptides (Leon et al., 2013. Conversely, a small study among healthy women found no significant increased risk (Srivastava et al., 2016).

The acute, short-term effect of heavy alcohol consumption is suggested by our finding that risk is increased among current heavy drinkers, as well as long-term heavy drinkers. When we defined ‘inconsistent drinkers’ according to their most recent level of intake instead of their modal level of intake, we only found higher levels of NT-proBNP among the ‘inconsistent but recently heavy group’. Further work is needed to demonstrate whether the effects of alcohol are reversible upon cessation of heavy drinking, but this finding highlights the need to have repeated data on both alcohol consumption and biomarkers (Bell et al., 2017a, 2017b) to unpack dynamics over time. Whilst we adjusted for major confounding factors, there is always a risk of residual confounding from unmeasured and unknown factors in observational studies.

Combined, our findings suggest heavy drinkers could be screened for NT-proBNP levels in order to identify those at high risk earlier in the clinical stages of heart failure and targeted for risk reduction strategies.

## Contributors

AB and SB developed the initial research idea. DO carried out the statistical analyses. DK was the principal investigator for the NSHD study. All Authors read the manuscript and approved submission.

## Funding

AB and DON were supported by UK Medical Research Council (MR/M006638/1), 10.13039/501100000269Economic and Social Research Council, and 10.13039/501100000280Alcohol Research UK. SB was funded by the National Institute for Health Research Blood and Transplant Research Unit in Donor Health and Genomics (NIHR BTRU-2014-10024). This work was supported by core funding from: the UK Medical Research Council (MR/L003120/1), the 10.13039/501100000274British Heart Foundation (RG/13/13/30194; RG/18/13/33946) and the National Institute for Health Research [Cambridge Biomedical Research Centre at the Cambridge University Hospitals NHS Foundation Trust]. The National Study for Health and Development is funded by the 10.13039/501100007155Medical Research Council (MC UU 1019/1).

## Declaration of Competing Interest

None declared
